# Old males reduce melanin‐pigmented traits and increase reproductive outcome under worse environmental conditions in common kestrels

**DOI:** 10.1002/ece3.1910

**Published:** 2016-01-27

**Authors:** David Lopez‐Idiaquez, Pablo Vergara, Juan Antonio Fargallo, Jesús Martinez‐Padilla

**Affiliations:** ^1^ Department of Evolutionary EcologyMuseo Nacional de Ciencias NaturalesMadridJosé Gutiérrez Abascal2. 23006MadridSpain; ^2^ Department of EcologyInstituto de Investigación en Recursos Cinegéticos – IRECRonda de Toledos/n. 13005Ciudad RealSpain; ^3^ Research Unit of Biodiversity (OU, CSIC, PA)Oviedo University33600MieresSpain

**Keywords:** Aging, longitudinal approach, long‐term monitoring, ornamentation, plumage coloration, sexual selection

## Abstract

Secondary sexual traits displayed by males and females may have evolved as a signal of individual quality. However, both individual quality and investment on producing or maintaining enhanced sexual traits change as individuals age. At the same time, the costs associated to produce sexual traits might be attenuated or increased if environmental conditions are benign or worse respectively. Accordingly, environmental conditions are expected to shape the association between the expression of sexual traits and their reproductive outcome as individuals age. Nonetheless, little is known about the environmental influence on the co‐variation between sexual traits and reproductive outcome throughout the life of individuals. We studied the age‐dependency of the number and size of back spots, a melanin‐based and sexual trait in adults of common kestrels (*Falco tinnunculus*). We analysed the age‐dependence of reproductive traits and the environmental influence, defined as vole abundance, using a 10‐year individual‐based dataset. We broke down age‐related changes of reproductive traits into within‐ and between‐individual variation to assess their contribution to population‐level patterns. Our results showed a within‐individual decrease in the number, but not the size, of back spots in males. The size of back spots was positively correlated with food availability in males. Reproductive performance of males increased as they aged, in agreement with the life‐history theory but depending of vole abundance. Remarkably, we found that having fewer back spots was positively associated with clutch size only for old individuals under low‐food conditions. We suggest that environmental variation may shape the association between the expression of a sexual signal and reproductive outcome. We speculate that the reliability of sexual traits is higher when environmental conditions are poor only for old individuals. Within an evolutionary context, we suggest that the expression of sexual traits might be constrained by environmental conditions at later stages of life.

## Introduction

Sexual selection is the main force driving the evolution and maintenance of secondary sexual traits (Darwin 1871; Andersson 1994). The reliability of these traits depends on the relative cost of production or maintenance, where only high‐quality individuals are able to produce and maintain them (Zahavi 1975; Grafen 1990; Kotiaho 2001). However, the expression of secondary sexual traits is not constant over individual lifespan. Senescence, understood as a within‐individual deterioration in physiological state over time, may arise because extrinsic mortality rates may reduce the force of selection in late life (Hamilton 1966; Partridge and Barton 1996). Within this life‐history context, individuals are expected to increase their investment in sexual signals as their reproductive value decreases to improve mating opportunities in the present (Kokko 1997). However, environmental variation plays a key role in natural and sexual selection, mediating the expression and reliability of sexual traits (Griffith et al. 1999; Vergara et al. 2012a,c) and the resolution of the trade‐off between ornament expression and other functions (Badyaev and Duckworth 2003; Martínez‐Padilla et al. 2010). Thus, environmental conditions may influence how individuals resolve the trade‐off between ornament expression and other costly functions depending on the life‐stage of individuals. Scientific literature has focused on different physiological or behavioral mechanisms that explain the variation of ornament expression, but we know rather little about how environmental variation influences the association between the expression of secondary sexual traits and reproductive parameters as individuals age.

In general, knowledge about how the expression of secondary sexual traits (SSTs) changes over the course of an individual's life is scarce, although there has been a recent increase in the number of studies on this topic (Forstmeier et al. 2006; Torres and Velando 2007; Galván and Møller 2009; Rivera‐Gutierrez et al. 2010; Velando et al. 2010). Nonetheless, the within‐ and between‐individual effects on age‐dependent variation in trait expression has been rarely teased apart (Evans et al. 2011; Evans and Sheldon 2013; Potti et al. 2013, 2014; Kervinen et al. 2015), and this is essential to understand whether trait variation over an individual's life is due to a genuine change in the mean within and among surviving individuals rather than selective disappearance or selective appearance of individuals in the population (Rebke et al. 2010). This lack of information is mainly due to the scarcity of long‐term and individual‐based data collection on the expression of traits in general and SSTs in particular (Nussey et al. 2008), raising the possibility that individual life‐history trajectories may remain hidden and unknown within the population variation (Vaupel and Yashin 1985; Bouwhuis et al. 2009; van de Pol and Wrigth 2009). In spite of the lack of studies disentangling the within‐ and between‐individual age effects in birds, some have tackled the issue from a within‐individual perspective (Evans et al. 2011; Evans and Sheldon 2013; Potti et al. 2013, 2014; Kervinen et al. 2015). Still the patterns showed on these studies are uncertain, while some show an increase of ornamentation as individuals age (Evans et al. 2011; Evans and Sheldon 2013; Potti et al. 2013), others find a lack of senescence on the expression of sexual traits (Candolin 2000a; Miller and Brooks 2005).

The association, however, between ornament expression and reproductive performance throughout the life of individuals is essential to understanding the evolution of sexual traits. According to sexual selection theory, an enhanced expression of sexual traits is expected to be associated with superior reproductive benefits, probably due to prime individual condition or genetic quality of the bearer. This association is maintained because producing or maintaining enhanced expression of sexual traits is traded‐off with other energetically demanding functions (Gustafsson et al. 1995; Badyaev and Duckworth 2003), like reproduction. As a general pattern, individuals at older ages usually reduce their reproductive value (Jones et al. 2008), old individuals favoring current mating opportunities, resulting in higher investment in sexual traits (Kokko 1997) as previously reported (Candolin 2000a,b). Thus, the study of age‐dependent effects on the expression of sexual traits requires a close examination of its link with reproductive parameters (Evans et al. 2011). Only two studies have explored the covariation between within‐individual age variation of sexual traits and reproduction in birds. In collared flycatchers (*Ficedula albicollis*), only females decreased their reproductive output while increasing the expression of their ornament as they aged (Evans et al. 2011). In pied flycatchers (*Ficedula hypoleuca*), females only decreased their reproductive output, though increased the expression of the trait, late in life if they expressed a forehead patch at early ages (first breeding attempt – Potti et al. 2013). Overall, these studies suggest that ornament expression with respect to age might be traded‐off with other resources, particularly with those allocated to reproduction. However, the sex‐dependent effect and few examples regarding within‐age expression of ornamental traits indicate a need for caution in generating accurate predictions within a life‐history context.

Crucial, however, is the notion that environmental conditions may change the intensity of sexual selection. In birds, examples of how the environment mediates the expression of sexual traits (Griffith et al. 1999; Chaine and Lyon 2008; Martínez‐Padilla et al. 2010, 2014; Robinson et al. 2012; Vergara and Martínez‐Padilla 2012; Vergara et al. 2012b,c), the condition‐dependence of their expression (Vergara et al. 2012a) and allocation priorities (Martínez‐Padilla et al. 2010, 2014; Vergara and Martínez‐Padilla 2012) are well‐known. From the recent scientific literature, it can be assumed that worse environmental conditions reduce the expression of ornamental traits (Cotton et al. 2004), and increase the reliability of sexual traits (Vergara et al. 2009, 2012a). Thus, only high‐quality individuals are able to express enhanced traits and reliably inform about individual quality under worse environmental conditions. Within a life‐history perspective, environmental conditions might also shape the abovementioned trade‐off between the investment in sexual traits and reproduction. Specifically, if environmental conditions are poor, individuals at older ages are expected to increase the investment in current mating opportunities and reproduction due to their reduced reproductive value. Otherwise, if environmental conditions are benign, the investment in producing an enhanced sexual trait and breeding outcome might be affordable for all individuals regardless of their age because resource abundance may allow them to confront these two energetically demanding functions.

Here, we aim to study within‐individual and lifetime variation of secondary sexual traits in both adult breeding male and female common kestrels (*Falco tinnunculus)*. Specifically, we explored how age covaries with reproductive parameters, and how environmental variation modulates this association in both males and females. We measured the number and size of black back spots in kestrel plumage, a trait that plays a role in sexual selection (Palokangas et al. 1994). Black coloration in kestrel plumage has been described to be based on melanin pigments (Fargallo et al. 2007a,b), showing both environmental and genetic components in its expression (Vergara et al. 2009; Kim et al. 2013). To explore the environmental influence, we used the abundance of common voles *Microtus arvalis* as a proxy of environmental variation, since the abundance of breeding kestrels in our population is sensitive to the interannual fluctuation of vole density (Fargallo et al. 2009). As a general prediction, we expect a stronger covariation between the expression of sexual traits and reproduction at older ages when environmental conditions are worse. We base our general prediction in three subsequent expectations that will be also explored: (1) within‐individual variation in the number and size of spots as individuals age; (2) covariation between ornamentation and reproductive performance; and (3) the association between environmental variation and ornament expression.

## Material and Methods

### Study species

The common kestrel (hereafter kestrel) is a medium‐sized raptor that exhibits marked sexual dimorphism in body size (females are 20% heavier than males) and in plumage coloration (Village 1990; Palokangas et al. 1994). Adult males range from brownish‐red to brick red with black spots on their backs and on the upper sides of their wings. Their heads range from completely brown to completely gray, and their rumps, upper tail‐coverts and tails are gray and mainly unbarred. Females are brown on the head, back and upperside of the wings, always with black spots. Juveniles show variable moulting of body feathers during their first year of life while maintaining most of their plumage during their first breeding season. Males and females differ in the expression of their back spots, with males showing less spots, mainly in adulthood (age ≥ 2). Back spots in males has been proved to have a role in sexual selection in kestrels (Palokangas et al. 1994). In barn owls (*Tyto alba*), females show a similar pattern of spottiness of a melanin‐pigmented trait (Roulin 2004), being a sexually selected trait that reflects genetic quality (Roulin 2004) and influence male mate choice (Roulin 1999), suggesting that spottiness might be sexually selected in females of our study species. Kestrel moult takes place usually after breeding (Village 1990). Because previous studies show that yearlings have a delayed plumage maturation (Village 1990; Vergara and Fargallo 2007), we only analysed adults (age ≥ 2) to avoid first‐year plumage (Evans et al. 2011).

### Study area

The study was conducted in the Campo Azálvaro region (40°40′N, 4°20′W), a homogeneous mountain grassland area in central Spain (1300 meters a.s.l) devoted mainly to cattle‐raising. Nest‐boxes were installed in the area progressively from 1994 to 2005, and the breeding population has been followed since then (Fargallo et al. 2001). During the period in which the study took place (2004–2013), there were a range of 24–45 kestrel pairs breeding each year (Fargallo et al. 2009).

### Data collection

This study took place between the breeding seasons of 2004 and 2013. Nests were monitored to detect laying date (the day that the first egg was laid), clutch size (mean = 5, range = 3–7, *n* = 277), and number of fledglings (mean = 4, range = 1–7, *n* = 277). Adult breeders were captured when nestlings were 10–13 days old (*n* = 277; males: *n* = 143; females: *n* = 134). At that time, body mass (to the nearest g), wing and tarsus length (to the nearest mm) were recorded, and a digital photograph was taken (see Supporting Information ‐ Nikon D70; objective: 18–70 mm AF‐S Nikkor DX). All photographs included the back and the right wing of every individual, along a scale that allowed us to determine the size of each trait in mm^2^. We determined whether they were yearlings (1 year old) or adults (≥2 years old) by using ring codes or plumage features (Vergara and Fargallo 2007).

### Ornament assessment

We measured the number and size of back spots in different areas of the wing and back of adult male and female kestrels using the photographs taken during their capture. The size of the back spots was the mean size value of five randomly selected spots using the “loop” tool of Photoshop CS5. Number and size of spots differed in their correlational level with the number and size of the spots in the other areas of the kestrel (see Data S1 Tables from S1 to S4 for further details) and they were not intercorrelated, either in males or in females (see Data S1 Tables S5 and S6 for further details). We also analysed the repeatability (Lessells and Boag 1987) of our spot size measure finding highly repeatable measurements only in the back spots (males *r* = 0.74; females *r* = 0.71; see Data S2‐Table S7 for further details). Thus, we decided to use only the number and size of back spots in the subsequent analyses.

### Environmental variables

The abundance of common voles was assessed by two trapping sessions per year. These were carried out in autumn and spring by setting out one hundred Sherman traps in four plots (25 each) for the length of the study. All traps were monitored six times for 3 days, three times in the early morning and three at sunset, during new moon periods to avoid the effects of moonlight on small mammal activity (Fargallo et al. 2009).

### Statistics and modeling

All analyses were carried out in R statistical software (packages lme4 and lmerTest ‐Bates et al. 2013; Kuznetsova et al. 2013). Kestrels show a remarkable sexual dimorphism in plumage (Village 1990), with males and females showing significant differences in both number (*F*_1,178_ = 312.5 *P* < 0.001) and size (*F*_1,178_ = 47.42, *P* < 0.001) of back spots. Thus, we analysed males and females separately in all models described below. In all statistical models, we followed a backwards‐stepwise selection procedure, in which all terms were initially included. Nonsignificant terms (*P* > 0.05) were removed sequentially.

#### Melanin‐based traits as proxies of individual quality

To establish the relationship between the ornamental traits and individual quality we performed Linear Mixed Models (LMMs). We first analysed the association between number and size of spots (dependent variables) and body mass or wing length in different models. Secondly, we explored the association between three reproductive variables (laying date, clutch size, and number of fledglings) and number and size of spots in different models. In this case, number and size of spots were included as explanatory variables and reproductive variables as dependent variables. In all models, age was included as a covariate, and individual identity and nest as random factors.

#### Age‐specific trait expression and reproductive performance

We first explored the cross‐sectional relationship between ornament expression and age. All color traits were z‐transformed (to mean = 0 and standard deviation = 1; z‐scores) to aid direct comparison across traits for each sex separately. We constructed Linear Mixed Models (LMM) with individual identity as a random factor. Age was fitted as a linear as well as quadratic effect and year as covariate (Conover and Schultz 1995; Conover et al. 2009).

Secondly, in order to explore within‐individual variation (WIA) on the expression of size and number of back spots, we developed longitudinal analyses, using a within‐subject centering approach to partition the population level age variation into within‐ and between‐individual effects (van de Pol and Wrigth 2009; Dingemanse and Dochtermann 2013). The within‐individual term is calculated by subtracting an individual's mean age from each individual age value (Within‐Individual Age – WIA = *x*_*ij*_ − *x*_*j*_^*′*^ *,* where *x*_*ij*_ is the age‐value of individual *j* at year *i,* and *x*_*j*_^*′*^ is the mean age of individual *j* in the dataset; van de Pol and Wrigth 2009). We also considered age at the last measurement (ALM) in order to take into account the effects of selective disappearance caused by an age‐mediated mortality pattern of individuals (van de Pol and Verhulst 2006). In order to control for the selective appearance of immigrant individuals coming from other populations with a different phenotypic expression, we considered the age of the first reproductive event of individuals in our population (AFM; van de Pol and Verhulst 2006). We constructed LMMs, considering number and size of spots (dependent variables) and ALM, AFM, and WIA as covariates. Individual identity was considered as a random factor.

We constructed LMMs with reproductive traits as dependent variables (laying date, clutch size, and number of fledglings) to explore the covariation between reproductive traits and ornamental traits. Lack of sample size did not allow us to explore the covariation between ornament expression and reproductive parameters in relation to age and environmental variation from a reaction norm perspective. To control for the possible effects of year in our models, ornamental and reproductive traits were z‐transformed for each year to aid direct comparison (see above). WIA, ALM, and AFM were included as independent variables. LMM models were fitted to a normal distribution of errors and individual identity as a random factor. We also included the interactions between the independent values. We included breeding seasons from 2004 to 2012 in the analyses, using the 2013 data to improve the accuracy of the ALM estimates.

#### Environmental variation

In order to assess the environmental influence on melanin‐based traits we used the number of voles in the previous spring (NVPS) capturing the food conditions that adult kestrels experienced at the time of moulting. We conducted LMMs for the number and size of spots as dependent variables and NVPS as explicative, individual identity was fitted as a random effect and individual age as a covariate. This analysis has a cross‐sectional perspective and does not allow us to tease apart the within‐ from the between‐individual effects of NVPS. From a longitudinal perspective, we grouped NVPS into five categories, according to the quartiles (NVPS_q_). We then used these categories to calculate a new variable (Within‐Individual “Vole”‐WIV = *x*_*ij*_− *x*_*j*_^*′*^ *,* where *x*_*ij*_ is the NVPS_q_ value of individual *j* at year *i,* and *x*_*j*_^*′*^ is the mean NVPS_q_ value of individual *j* in the dataset), analogous to WIA, that allowed us to tease apart the within‐individual effects of the environment. We included this variable as explicative in order to explain the variation in the number and size of back spots.

In order to explore the effect of environmental variation on the relationship between age and reproduction, we categorized NVPS into high and low if annual values were above or below the overall mean (NVPS_c_, coded as “high” or “low”). Due to sample size limitations, we also categorized WIA (WIA_c_) of each individual in three levels as −1, 0 and 1, representing early ages (−1: WIA_c_ < 0), midlife ages (0: WIA_c_ = 0), and late life (1: WIA_c_ > 0). Melanin‐based traits were z‐transformed for the categories of NVSP_c_ and WIA_c_. NVPS_c_, WIA_c_ and the z‐scores of the number and size of spots were the explanatory variables in our models. Clutch size and number of offspring were considered proxies of fitness and were mean‐centered for each category of NVSP_c_ and WIA_c_, and included as dependent variables in our models. We tested the interaction between NVPS_c_, WIA_c_ and ornament expression in explaining variation of clutch size or number of offspring. The models included year and individual identity as random effects and were carried out separately for the number and size of spots and for males and females.

## Results

### Melanin‐based traits as proxies of individual quality

We did not find any association between wing length on the expression of the studied melanin‐based traits, either for males (*P* > 0.153) or for females (*P* > 0.208). However, we found that spot size was positively correlated to body mass in females (0.082 ± 0.038, *F*_1,57.86_ = 4.47, *P* = 0.039) but not in males (*F*_1,97.59_ = 0.00007, *P* = 0.993). No significant relationship was found between spot number and body mass, either in females (*F*_1,58.9_ = 0.45, *P* = 0.505) or in males (*F*_1,95.28_ = 0.176, *P* = 0.675).

In males, there was a significant negative relationship between spot size and laying date (estimate = −0.474 ± 0.202, *F*_1,90.09_ = 5.469, *P* = 0.021). However, spot size was not correlated with clutch size (*F*_1,97.65_ = 7.936, *P* = 0.407) and marginally positively with number of fledglings (0.058 ± 0.030 *F*_1,88.49_ = 3.727, *P* = 0.056). No significant relationship was found between number of spots and the reproductive variables (*P* > 0.408). In females our results show that laying date was positively correlated with number of spots (0.148 ± 0.067, *F*_1,51.04_ = 4.783, *P* = 0.033) and negatively with size of spots (−0.470 ± 0.195, *F*_1,60.59_ = 5.807, *P* = 0.019). No significant relationships were found for clutch size or number of fledglings neither with spot number (CS: *F*_1,67.09_ = 0.042, *P* = 0.837, NF: *F*_1,53.97_ = 0.271, *P* = 0.604) or spot size (CS: *F*_1,69.72_ = 0.242, *P* = 0.624, NF: *F*_1,72.1_ = 2.849, *P* = 0.095).

### Cross‐sectional patterns of ornamental trait change

The cross‐sectional analyses showed a significant negative relationship between age and number of back spots in males but not in females (Table 1). We also found that age was not a significant predictor of the size of back spots, either in males or in females (Table 1). Year was retained as a significant negative predictor for the number of spots in males (Table 1).

**Table 1:** Results of cross‐sectional models of expression of melanin‐based traits in male and female kestrels aged ≥ 2. Variables included in the final model are in bold; values for excluded variables refer to the step before their exclusion (E. Seq.)

Parameter	Estimate	SE	*F*	*P*	E. Seq.	Estimate	SE	*F*	*P*	E. Seq.
	Male number of back spots (*n* = 105)					Female number of back spots (*n* = 75)				
Age	**−0.156**	**0.067**	***F***_**1,32**_ ** = 5.354**	**0.027**		0.028	0.065	*F*_1,30_ = 0.193	0.663	
Age2	−0.006	0.042	*F*_1,31_ = 0.023	0.879	1	−0.030	0.030	*F*_1,29_ = 1.001	0.325	2
Year	**−0.129**	**0.039**	***F***_**1,32**_ ** = 10.92**	**0.002**		0.014	0.052	*F*_1,28_ = 0.021	0.787	1
	Male size of back spots (*n* = 105)					Female size of back spots (*n* = 75)				
Age	0.008	0.074	*F*_1,33_ = 0.012	0.911		−0.014	0.069	*F*_1,30_ = 0.045	0.832	
Age2	−0.059	0.046	*F*_1,32_ = 1.631	0.210	2	−0.022	0.030	*F*_1,29_ = 0.537	0.469	2
Year	0.026	0.045	*F*_1,31_ = 0.335	0.566	1	0.029	0.055	*F*_1,28_ = 0.223	0.594	1

### Within‐individual patterns of ornament and reproductive change

**Figure 1:**
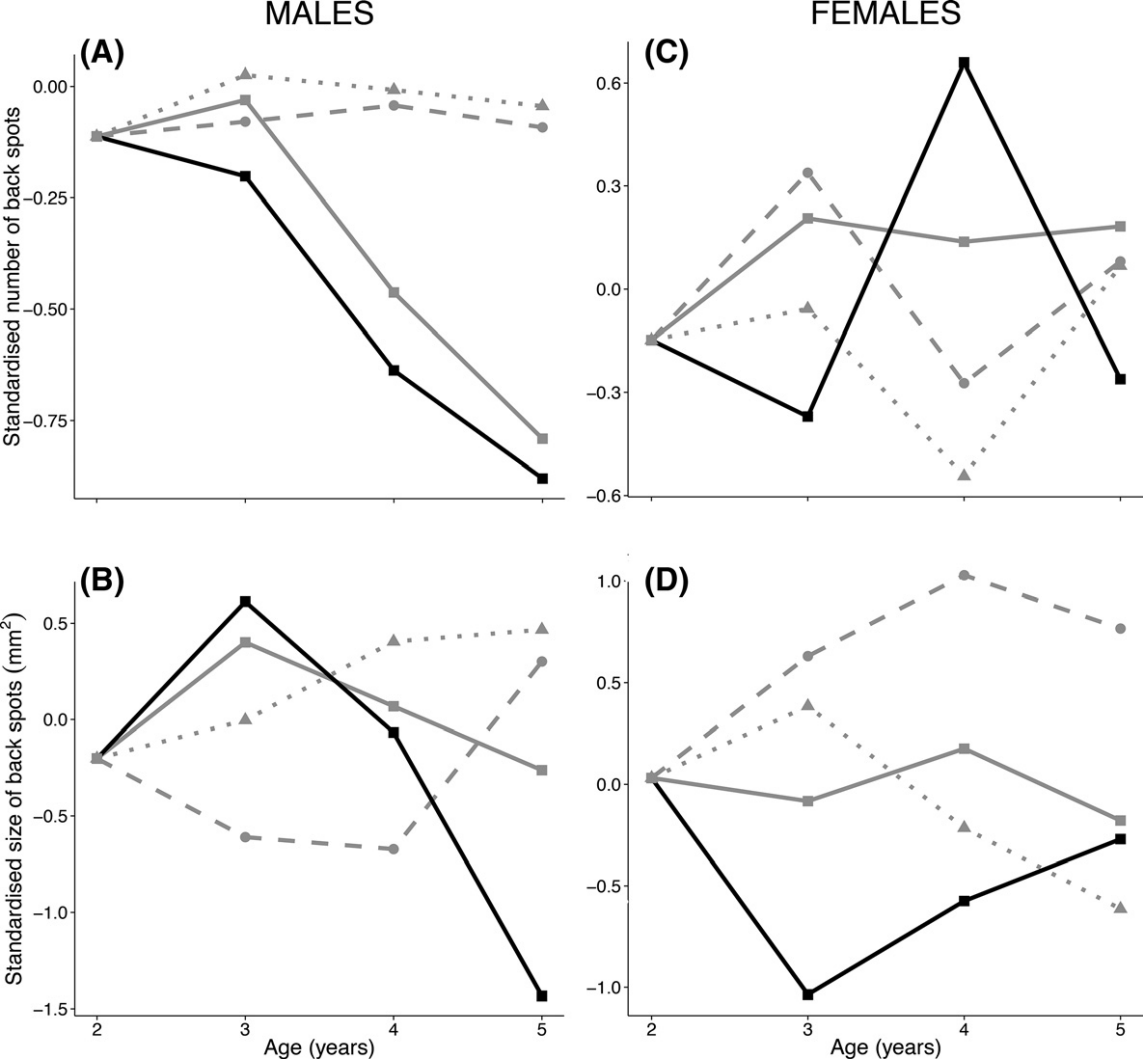
Patterns of standardized melanin‐based trait expression for males (A, B) and females (C, D) of known age (≥2 years). The thick black line with squares represents within‐individual change. The thick gray line with squares represents the population level pattern. The dashed light gray with circles line shows the contribution of the selective appearance and the spotted gray line with triangles shows the contribution of the selective disappearance to the population‐level mean. Note that the scale may differ between graphs. To perform these graphics we followed Rebke et al. (2010). We only represented age of birds up to 5 years old because of the limited sample size of individuals at higher ages classes (Age 6 = 4, 7 = 1 and 8 = 1; see Data S3 Table S8 for further details).

We found that WIA was a significant predictor of the number back spots in males (Table 2, Fig. 1). No significant relationship was found for the size of back spots in males or females (Table 2). Regarding reproductive performance in males, we found that the number of fledglings was positively correlated with ALM and marginally with WIA (Table 3). We also found a significant relationship between laying date and ALM (Table 3). Finally, we found that WIA and AFM, and their interaction were significant and positive predictors of clutch size. In females, we found no significant relationship with any of the studied variables (Tables 2 and 3).

**Table 2:** Results of individual‐level models of expression of age‐dependent plumage melanin in male and female kestrels of known age (≥2). The annual mean expression level was subtracted from each trait measurement to control for population‐level variation in ornamentation, before standardization (see M&M for further details). Within‐individual age (WIA) represents the individual change during the life of a given individual. Age at measurement (ALM) is a proxy of longevity. Age at first measurement (AFM) represents the first reproduction in our population. Variables included in the final model are in bold; values for excluded variables refer to the step before their exclusion (E. Seq.)

Parameter	Estimate	SE	*F*	*P*	E. Seq.	Estimate	SE	*F*	*P*	E. Seq.
	Male number of back spots (*n* = 105)					Female number of back spots (*n* = 75)				
***WIA***	**−0.289**	**0.111**	***F***_**1,33**_ ** = 6.803**	**0.013**		−0.051	0.121	*F*_1,30_ = 0.026	0.675	5
*AFM*	−0.106	0.113	*F*_1,68_ = 0.708	0.348	3	−0.133	0.184	*F*_1,41_ = 0.745	0.475	3
*ALM*	−0.074	0.072	*F*_1,69_ = 1.076	0.303	5	0.039	0.054	*F*_1,42_ = 0.535	0.648	
WIA*AFM	0.059	0.185	*F*_1,31_ = 0.060	0.798	1	−0.034	0.201	*F*_1,28_ = 0.693	0.863	2
WIA*ALM	−0.089	0.086	*F*_1,32_ = 1.077	0.307	4	−0.100	0.056	*F*_1,29_ = 3.181	0.085	4
ALM*AFM	0.035	0.070	*F*_1,67_ = 0.252	0.616	2	0.018	0.113	*F*_1,40_ = 0.027	0.869	1
	Male size of back spots (*n* = 105)					Female size of back spots (*n* = 75)				
*WIA*	−0.091	0.115	*F*_1,33_ = 0.476	0.433	5	0.007	0.110	*F*_1,30_ = 0.002	0.947	5
*AFM*	−0.072	0.109	*F*_1,68_ = 0.023	0.531	3	0.022	0.180	*F*_1,42_ = 0.015	0.901	
*ALM*	0.070	0.071	*F*_1,69_ = 0.966	0.329		−0.025	0.076	*F*_1,41_ = 0.150	0.739	3
WIA*AFM	−0.020	0.198	*F*_1,31_ = 0.092	0.919	1	−0.243	0.164	*F*_1,29_ = 2.197	0.149	4
WIA*ALM	−0.115	0.087	*F*_1,32_ = 1.747	0.195	4	0.053	0.063	*F*_1,28_ = 0.002	0.412	1
ALM*AFM	−0.027	0.069	*F*_1,67_ = 0.154	0.695	2	0.110	0.120	*F*_1,40_ = 0.842	0.364	2

**Table 3:** Results of individual‐level models of the reproductive traits in male and female kestrels of known age (≥2). The annual mean expression level was subtracted from each trait measurement to control for population‐wide variation in ornamentation, before standardization by z‐transformation. Variables included in the final model are in bold; values for excluded variables refer to the step before their exclusion (E. Seq.)

Parameter	Estimate	SE	*F*	*P*	E. Seq.	Estimate	SE	*F*	*P*	E. Seq.
	(Male) Number of fledglings (*n* = 109)					(Female) Number of fledglings (*n* = 75)				
*WIA*	0.297	0.153	*F*_1,35_ = 4.486	0.061		0.011	0.116	*F*_1,30_ = 0.0002	0.921	5
*AFM*	0.082	0.116	*F*_1,70_ = 3.469	0.481	4	0.097	0.163	*F*_1,42_ = 0.535	0.555	
*ALM*	0.189	0.075	*F*_1,71_ = 6.270	**0.014**		−0.006	0.062	*F*_1,41_ = 0.051	0.912	3
WIA*ALM	−0.287	0.263	*F*_1,34_ = 0.502	0.282	2	−0.263	0.178	*F*_1,29_ = 2.182	0.150	4
WIA*AFM	−0.011	0.113	*F*_1,33_ = 0.157	0.918	1	−0.021	0.068	*F*_1,28_ = 0.103	0.750	2
ALM*AFM	−0.129	0.076	*F*_1,69_ = 2.918	0.092	3	0.0005	0.113	*F*_1,40_ = 0.00002	0.996	1
	(Male) Laying date (*n* = 109)					(Female) Laying date (*n* = 75)				
*WIA*	−0.090	0.131	*F*_1,35_ = 0.533	0.497	5	−0.1463	0.1092	*F*_1,30_ = 0.524	0.190	4
*AFM*	−0.100	0.100	*F*_1,70_ = 3.752	0.322	3	−0.2414	0.187	*F*_1,41_ = 0.917	0.204	5
*ALM*	−0.146	0.064	*F*_1,71_ = 5.131	**0.026**		0.069	0.066	*F*_1,42_ = 1.115	0.297	
WIA*ALM	0.187	0.218	*F*_1,33_ = 0.367	0.379	2	0.1956	0.156	*F*_1,29_ = 1.563	0.221	3
WIA*AFM	−0.106	0.090	*F*_1,34_ = 1.369	0.250	4	−0.085	0.059	*F*_1,28_ = 0.565	0.158	2
ALM*AFM	−0.0007	0.069	*F*_1,69_ = 0.0001	0.991	1	0.059	0.118	*F*_1,40_ = 0.246	0.622	1
	(Male) Clutch size (*n* = 109)					(Female) Clutch size (*n* = 75)				
WIA	0.975	0.352	*F*_1,34_ = 4.618	**0.009**		−0.139	0.109	*F*_1,30_ = 1.622	0.212	
AFM	0.202	0.088	*F*_1,71_ = 4.165	**0.024**		0.101	0.172	*F*_1,42_ = 0.344	0.560	5
ALM	0.083	0.087	*F*_1,70_ = 1.044	0.342	1	0.007	0.067	*F*_1,41_ = 0.0009	0.907	3
WIA*ALM	−0.371	0.169	*F*_1,34_ = 4.774	**0.035**		−0.317	0.166	*F*_1,29_ = 3.630	0.066	4
WIA*AFM	0.037	0.086	*F*_1,33_ = 0.343	0.670	1	−0.033	0.064	*F*_1,28_ = 0.273	0.605	2
ALM*AFM	−0.045	0.065	*F*_1,69_ = 0.471	0.494	2	−0.053	0.113	*F*_1,40_ = 0.236	0.629	1

### Association between environmental variation and ornamental traits

**Figure 2:**
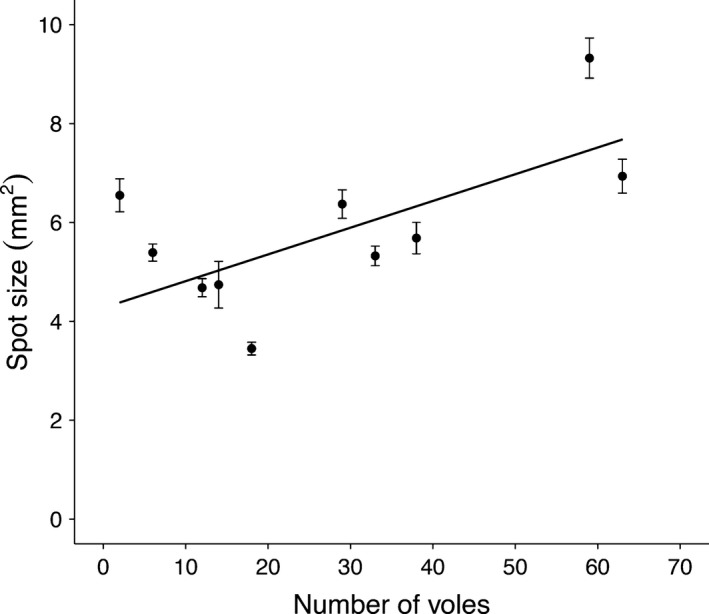
Positive cross‐sectional pattern of vole abundance of previous spring (NVPS) on back spot size (mean ± SD) of male kestrels of known age (≥2 years).

The cross‐sectional approach showed a significant and positive relationship between NVPS_q_ and the size of male back spots (estimate: 0.011 ± 0.004; *F*_1,33_ = 6.353 *P* = 0.016 – Fig. 2). We then analysed the relationship between the within‐individual effect of voles (WIV) and the melanin‐based traits. We found a positive association between the WIV and the size of male back spots (estimate: 0.198 ± 0.091; *F*_1,33_ = 4.761 *P* = 0.036). No other significant relationships were found in males or females (*P* > 0.079).

### Environmental influence on the association between reproduction and ornament expression

**Figure 3:**
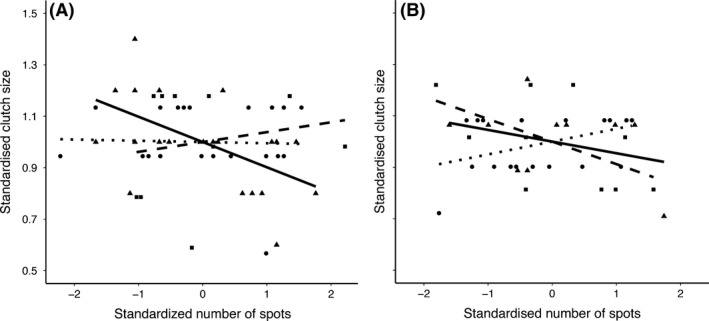
Relationship between clutch size and number of spots of known age of males of common kestrel under low (A) and high (B) vole abundance. The lines represent individuals at three different stages of life: Early life (short dashed line and black squares), mid‐life (dotted line and black circles), and late life (black line and black triangles). See methods for further details.

In males, we found that the association between clutch size and number of back spots was mediated by WIA_c_ and NVPS_c_ (interaction number of back spots*WIA_c_*NVPS_c_; *F*_2,82.337_ = 3.949, *P* = 0.023). We split this triple interaction and explored the relationship between clutch size and number of spots under the high and low food availability scenarios. In a low food scenario, we found that the significant relationship between clutch size and number of spots was mediated by WIA_c_ (interaction number of back spots*WIA_c_; *F*_2,45.45_ = 3.315; *P* = 0.045; Fig. 3). In particular, we found a negative association between clutch size and number of back spots only in individuals at later stages of life (WIA_c_ > 0 estimate = −0.098 ± 0.033, *F*_1,21_ = 8.796 *P* = 0.007; *P* > 0,582 for WIA_c_ = 0 and WIA_c_ < 0; Fig. 3). Under a high food availability context, our results also show a significant influence of WIA_c_ in the relationship between the clutch size and the number of spots (F_2, 5.014_ = 10.563, *P* = 0.015). Specifically, we found a significant association between clutch size and number of back spots only in individuals at midlife (WIA_c_ = 0: 0.049 ± 0.019 *F*_1,22_ = 6.221 *P* = 0.020). We also found a marginal tendency for early life individuals (WIA_c_ < 0: −0.087 ± 0.042, *F*_1,10_ = 4.204, *P* = 0.067) and no significant relationship for those in age class 3 (WIA_c_ > 0 *P* = 0.0344). Number of fledglings and number of back spots were not mediated by WIA_c_ and NVPS_c_ (interaction number of back spots*WIA_c_*NVPS_c_; *F*_2,93_ = 0.398, *P* = 0.692).

Regarding size of back spots, we did not find any mediating effect of WIA_c_ or NVPS_c_ on any proxy of fitness (clutch size: interaction size of back spots*WIA_c_*NVPS_c_; *F*_2,83.163_ = 2.943, *P* = 0.058; number of fledglings: number of back spots*WIA_c_*NVPS_c_; *F*_2,92.356_ = 2.686, *P* = 0.073). We did not find any relationship in females (*P* > 0.177).

## Discussion

We used a 10‐year dataset to investigate the influence of age and environmental variation on the expression of ornamental melanin‐based traits and compared it with aging patterns of reproductive characters. Cross‐sectional and within‐individual analyses show that number, but not size, of spots decreases as individuals age, only in males. This suggests that the number of spots may act as an index of quality as individuals that live longer show less spots, providing a proxy of quality and perhaps fitness benefits for females (Kokko and Lindstrom 1996; Kokko 1998; Brooks and Kemp 2001). This is partially supported by our results, where we found that older males produce larger clutch sizes and raise more offspring. In females, back spots may be a reliable index of individual quality, since females showing more and larger spots had earlier laying dates.

Interestingly, we found an increase in reproductive performance with age in males, as predicted by life‐history theory (Williams 1966). Specifically, clutch size increases as males age and is larger for individuals whose first breeding event is earlier in their lives. In addition, brood size also increases as individuals age and is larger for individuals that live longer. This is consistent with the idea that by mating with older males, females may obtain more fitness benefits than by mating with younger ones (Kokko and Lindstrom 1996; Kokko 1998; Brooks and Kemp 2001). Regarding the association between reproductive output and number of spots, our results fit within a life‐history context and disagree with the senescence‐based scenario, which predicts a decrease in reproductive performance throughout life. We cannot rule out the possibility that individuals may show such senescence patterns in reproduction at older ages, undetected here either because of a deficit in the number of older individuals or a stronger selection in this species against senescent individuals.

Our results suggest that environmental circumstances may drive the association between trait expression and fitness, considering clutch size as a proxy, at different life stages. Our results reveal that number of spots and clutch size are negatively correlated only for individuals in later periods of life under a scenario of low food availability. Considering the association between fitness and number of back spots as an individual index of quality, females will obtain reproductive benefits when choosing older (less spotted) individuals as mates. But this benefit will increase under worse environmental conditions, which is in our case low food abundance. Under this scenario and supporting our general prediction, the reliability of the signal increases when environmental conditions worsen and particularly for old individuals. This may explain why individuals decrease their number of back spots at older ages. Within an evolutionary context, it is possible that environmental fluctuation may prevent a change in the frequency distribution of the number of back spots in males in our population since high or low food quality scenarios fluctuate, although not cyclically, favoring fluctuating selection. These results suggest that environmental conditions may shape the strength of selection on ornamental traits during different stages of an individual's life. Unfortunately, specific analyses studying the selection of the covariation between ornamental traits and reproduction in relation to aging and environmental conditions are needed to confirm this pattern. Our results also show that under benign environmental conditions middle‐aged individuals with high number of spots have larger clutch sizes. These results may explain the variance in the expression of number of spots in different individuals, as they may gain mating benefits depending on the environmental conditions.

It is common in the study of the function or evolutionary dynamics of sexual traits to consider the size or coloration of given traits, but little is known when exploring the expression of different characteristics of the same trait. We found that number but not size of back spots changes throughout life and that size but not number is influenced by food availability. The size of back spots changes in accordance to the environmental situations that individuals face during their lives. A key environmental feature that explains the size of spots in male breeding kestrels appears to be the abundance of common voles, a key prey for kestrels in our population (Navarro‐López et al. 2014). This result agrees with the idea of environmental influence mediating the expression of melanin‐based traits (Fargallo et al. 2007a,b; Vergara et al. 2009) even when they have a strong genetic influence in kestrels (Kim et al. 2013). We suggest that the size of back spots may provide information about male food intake during moult, as they may influence the hormonal production that regulates melanogenesis (Jawor and Breitwisch 2003).

In spite of previous studies showing that melanin‐based coloration is under genetic control (Niecke et al. 2003; Roulin and Dijkstra 2003; Griffith et al. 2006; Kim et al. 2013), the role of environmental variance in the expression of melanin‐based traits is striking (Fargallo et al. 2007b; Vergara et al. 2009; Kim et al. 2013). Our results suggest that the size of back spots might be a reliable index of a male's ability to obtain high‐quality territories in terms of food availability, while the expression of the number of back spots might be more associated with nonenvironmental sources of variation. Therefore, two characteristics of the same melanin‐based trait can be driven by different factors. Perhaps additive genetic variance explains a higher proportion of total variance of the expression of number of spots in males, while food abundance drives the size of back spots. Regardless of the specific factors explaining the variance of the expression of these two characteristics, it seems plausible to think that these two characteristics may work in a multiple message context, indicating the different characteristics of individuals (Møller and Pomiankowski 1993; Candolin 2003; Chaine and Lyon 2008). On the one hand, the number of spots may convey information about the genetic quality of the individuals, and on the other hand, the size of back spots may provide information about males' skills for obtaining good territories during moult.

Our study also reveals a sex‐dependent variation in the expression of back spots and reproductive output. Reasons for such variation are uncertain but raise a key question in associating age‐dependent variation of melanin‐based traits with sexual dimorphism. Females change the expression of melanin‐based traits very little throughout their lives in comparison to males. Specifically, males do change their phenotype showing a less young‐like phenotype (less spots), increasing sexual dimorphism as individuals age. This agrees with our results where we found a positive selection on more experienced males with fewer back spots, an effect that becomes stronger in worse environmental (low food) conditions. A potential covariation between an age‐dependent expression of melanic traits and reproduction in males, but not in females, may potentially provide new venues for the study of the evolution of sexual dimorphism in the expession of melanin‐pigmented traits.

Overall, our study suggests a sex‐dependent effect of melanin‐based back coloration where there are within‐individual decreases in the number of back spots only in males. Within‐age decreases in the number of back spots and the increase in reproductive performance in males as they age is in agreement with a life‐history perspective but not so with the senescence context. Finally, our results point toward an environment‐mediated selection on the expression of phenotypes particularly stronger in later stages of life. We suggest that environmentally and genetically driven selection forces may act differentially on two characteristics of the same melanin‐based trait, explaining the expression of the same trait.

## Conflict of Interest

None declared.

## Supporting information


**Data S1.** Inter‐relationship melanin‐based measured traits.
**Table S1.** Results of LMM analysing the inter‐relationship of the spot number in four patches of male common kestrels (*Falco tinnunculus*).
**Table S2.** Results of LMM with normal errors analysing the inter‐relationship of the spot size in four patches of male common kestrels (*Falco tinnunculus*).
**Table S3.** Results of LMM analysing the inter‐relationship of the spot number in four patches of female common kestrels (*Falco tinnunculus*).
**Table S4.** Results of GLMM with normal errors analysing the inter‐relationship of the spot size in four patches of female common kestrels (*Falco tinnunculus*).
**Table S5.** Results of the LMM analysing the relationship between the number and size of the spots in each patch in male common kestrels (*Falco tinnunculus*).
**Table S6.** Results of the LMM analysing the relationship between the number and size of the spots in each patch in female common kestrels (*Falco tinnunculus*).
**Figure S1.** Common kestrel dorsum divided in four areas: (a) back, (b) auxiliary feathers (c) greater coverts and (d) median and lesser coverts.
**Data S2.** Measured trait repeatability.
**Table S7.** Results of the repeatability analysis done for the size of the measured traits in both males and females.
**Data S3.** Number of observations for males and females in each age class.
**Table S8.** Number of observations on each age class in males and females.

## References

[ece31910-bib-0001] Andersson, M. 1994 Sexual selection. Princeton Univ. Press, Princeton, NJ.

[ece31910-bib-0002] Badyaev, A. V. , and R. A. Duckworth . 2003 Context‐dependent sexual advertisement: plasticity in development of sexual ornamentation throughout the lifetime of a passerine bird. J. Evol. Biol. 16:1065–1076. 14640398 10.1046/j.1420-9101.2003.00628.x

[ece31910-bib-0003] Bates, D. , M. Maechler , and B. M. Bolker . 2013 lme4: Linear mixed‐effects models using S4 classes. R package version 0.999999‐2.

[ece31910-bib-0004] Bouwhuis, S. , B. C. Sheldon , S. Verhulst , and A. Charmantier . 2009 Great tits growing old: selective disappearance and the partitioning of senescence to stages within the breeding cycle. Proc. Biol. Sci. 276:2769–2777. 19403537 10.1098/rspb.2009.0457 PMC2839957

[ece31910-bib-0005] Brooks, R. , and D. J. Kemp . 2001 Can older males deliver the good genes? Trends Ecol. Evol. 16:308–313. 11369109 10.1016/s0169-5347(01)02147-4

[ece31910-bib-0006] Candolin, U. 2000a Changes in expression and honesty of sexual signalling over the reproductive lifetime of sticklebacks. Proc. R. Soc. Lond. B Biol. Sci. 267:2425–2430. 10.1098/rspb.2000.1301 PMC1690825 11133033

[ece31910-bib-0007] Candolin, U. 2000b Increased signalling effort when survival prospects decrease: male–male competition ensures honesty. Anim. Behav. 60:417–422. 11032643 10.1006/anbe.2000.1481

[ece31910-bib-0008] Candolin, U. 2003 The use of multiple cues in mate choice. Biol. Rev. 78:575–595. 14700392 10.1017/s1464793103006158

[ece31910-bib-0009] Chaine, A. S. , and B. E. Lyon . 2008 Adaptive plasticity in female mate choice dampens sexual selection on male ornaments in the lark bunting. Science 319:459–462. 18218896 10.1126/science.1149167

[ece31910-bib-0010] Conover, D. O. , and E. T. Schultz . 1995 Phenotypic similarity and the evolutionary significance of countergradient variation. Trends Ecol. Evol. 10:248–252. 21237029 10.1016/S0169-5347(00)89081-3

[ece31910-bib-0011] Conover, D. O. , T. A. Duffy , and L. A. Hice . 2009 The covariance between genetic and environmental influences across ecological gradients. Ann. N. Y. Acad. Sci. 1168:100–129. 19566705 10.1111/j.1749-6632.2009.04575.x

[ece31910-bib-0012] Cotton, S. , K. Fowler , and A. Pomiankowski . 2004 Do sexual ornaments demonstrate heightened condition‐dependent expression as predicted by the handicap hypothesis? Proc. R. Soc. Lond. B Biol. Sci. 271:771–783. 10.1098/rspb.2004.2688 PMC1691662 15255094

[ece31910-bib-0013] Darwin, C. 1871 The descent of the man and selection in relation to sex. Murray, London, UK.

[ece31910-bib-0014] Dingemanse, N. J. , and N. A. Dochtermann . 2013 Quantifying individual variation in behaviour: mixed‐effect modelling approaches. J. Anim. Ecol. 82:39–54. 23171297 10.1111/1365-2656.12013

[ece31910-bib-0015] Evans, S. R. , and B. C. Sheldon . 2013 Pigments versus structure: examining the mechanism of age‐dependent change in a carotenoid‐based colour. J. Anim. Ecol. 82:418–428. 23194384 10.1111/1365-2656.12008

[ece31910-bib-0016] Evans, S. R. , L. Gustafsson , and B. C. Sheldon . 2011 Divergent patterns of age‐dependece in ornamental and reproductive traits in the collared flycaycher. Evolution 65:1623–1636. 21644953 10.1111/j.1558-5646.2011.01253.x

[ece31910-bib-0017] Fargallo, J. A. , G. Blanco , J. Potti , and J. Viñuela . 2001 Nestbox provisioning in a rural population of Eurasian Kestrels: breeding performance, nest predation and parasitism. Bird Study 48:236–244.

[ece31910-bib-0018] Fargallo, J. A. , T. Laaksonen , E. Korpimäki , and K. Wakamatsu . 2007a A melanin‐based trait reflects environmental growth conditions of nestling male Eurasian kestrels. Evol. Ecol. 21:157–171.

[ece31910-bib-0019] Fargallo, J. A. , J. Martínez‐Padilla , A. Toledano‐Díaz , J. Santiago‐Moreno , and J. A. Dávila . 2007b Sex and testosterone effects on growth, immunity and melanin coloration of nestling Eurasian kestrels. J. Anim. Ecol. 76:201–209. 17184369 10.1111/j.1365-2656.2006.01193.x

[ece31910-bib-0020] Fargallo, J. A. , J. Martínez‐Padilla , J. Viñuela , G. Blanco , I. Torre , P. Vergara , et al. 2009 Kestrel‐prey dynamic in a mediterranean region: the effect of generalist predation and climatic factors. PLoS ONE 4:e4311. 19234618 10.1371/journal.pone.0004311 PMC2645439

[ece31910-bib-0021] Forstmeier, W. , D. Hasselquist , S. Bensch , and B. Leisler . 2006 Does song reflect age and viability? A comparison between two populations of the great reed warbler *Acrocephalus arundinaceus* . Behav. Ecol. Sociobiol. 59:634–643.

[ece31910-bib-0022] Galván, I. , and A. P. Møller . 2009 Different roles of natural and sexual selection on senescence of plumage colour in the barn swallow. Funct. Ecol. 23:302–309.

[ece31910-bib-0023] Grafen, A. 1990 Biological signals as handicaps. J. Theor. Biol. 144:517–546. 2402153 10.1016/s0022-5193(05)80088-8

[ece31910-bib-0024] Griffith, S. C. , I. P. F. Owens , and T. Burke . 1999 Enviromental determination of a sexually selected trait. Nature 400:358–360.

[ece31910-bib-0025] Griffith, S. C. , T. H. Parker , and V. A. Olson . 2006 Melanin‐ versus carotenoid‐based sexual signals: is the difference really so black and red? Anim. Behav. 71:749–763.

[ece31910-bib-0026] Gustafsson, L. , A. Qvarnstrom , and B. C. Sheldon . 1995 Trade‐offs between life‐history traits and a secondary sexual character in male collared flycatchers. Nature 375:311–313.

[ece31910-bib-0027] Hamilton, W. D. 1966 The moulding of senescence by natural selection. J. Theor. Biol. 12:12–45. 6015424 10.1016/0022-5193(66)90184-6

[ece31910-bib-0028] Jawor, J. M. , and R. Breitwisch . 2003 Melanin ornaments, honesty and sexual selection. Auk 120:249–265.

[ece31910-bib-0029] Jones, O. R. , J.‐M. Gaillard , S. Tuljapurkar , J. S. Alho , K. B. Armitage , P. H. Becker , et al. 2008 Senescence rates are determined by ranking on the fast–slow life‐history continuum. Ecol. Lett. 11:664–673. 18445028 10.1111/j.1461-0248.2008.01187.x

[ece31910-bib-0030] Kervinen, M. , C. Lebigre , R. V. Alatalo , H. Siitari , and C. D. Soulsbury . 2015 Life‐history differences in age‐dependent expressions of multiple ornaments and behaviors in a lekking bird. Am. Nat. 185:13–27. 25560550 10.1086/679012

[ece31910-bib-0031] Kim, S. Y. , J. A. Fargallo , P. Vergara , and J. Martinez‐Padilla . 2013 Multivariate heredity of melanin‐based coloration, body mass and immunity. Heredity 111:139–146. 23591519 10.1038/hdy.2013.29 PMC3716269

[ece31910-bib-0032] Kokko, H. 1997 Evolutionarily stable strategies of age‐dependent sexual advertisement. Behav. Ecol. Sociobiol. 41:99–107.

[ece31910-bib-0033] Kokko, H. 1998 Good genes, old age and life‐history trade‐offs. Evol. Ecol. 12:739–750.

[ece31910-bib-0034] Kokko, H. , and J. Lindstrom . 1996 Evolution of female preference for old mates. Proc. Biol. Sci. 263:1533–1538.

[ece31910-bib-0035] Kotiaho, J. S. 2001 Costs of sexual traits: a mismatch between theoretical considerations and empirical evidence. Biol. Rev. 76:365–376. 11569789 10.1017/s1464793101005711

[ece31910-bib-0036] Kuznetsova, A. , P. B. Brockhoff , and R. H. B. Christensen . 2013 lmerTest:Test for random and fixed effects for linear mixed effect models (lmer objects of lme4 package).

[ece31910-bib-0037] Lessells, C. M. , and P. T. Boag . 1987 Unrepeatable repeatabilities a common mistake. Auk 104:116–121.

[ece31910-bib-0038] Martínez‐Padilla, J. , F. Mougeot , L. M. I. Webster , L. Pérez‐Rodríguez , and S. B. Piertney . 2010 Testing the interactive effects of testosterone and parasites on carotenoid‐based ornamentation in a wild bird. J. Evol. Biol. 23:902–913. 20536879 10.1111/j.1420-9101.2010.01956.x

[ece31910-bib-0039] Martínez‐Padilla, J. , L. Pérez‐Rodríguez , F. Mougeot , S. Ludwig , and S. M. Redpath . 2014 Intra‐sexual competition alters the relationship between testosterone and ornament expression in a wild territorial bird. Horm. Behav. 65:435–444. 24698833 10.1016/j.yhbeh.2014.03.012

[ece31910-bib-0040] Miller, L. K. , and R. Brooks . 2005 The effects of genotype, age, and social environment on male ornamentation, mation behaviour and attractiveness. Evolution 59:2414–2425. 16396182

[ece31910-bib-0041] Møller, A. P. , and A. Pomiankowski . 1993 Why have birds got multiple sexual ornaments? Behav. Ecol. Sociobiol. 32:167–176.

[ece31910-bib-0042] Navarro‐López, J. , P. Vergara , and J. A. Fargallo . 2014 Trophic niche width, offspring condition and immunity in a raptor species. Oecologia 174:1215–1224. 24368708 10.1007/s00442-013-2855-9

[ece31910-bib-0043] Niecke, M. , S. Rothlaender , and A. Roulin . 2003 Why do melanin ornaments signal individual quality? Insights from metal element analysis of barn owl feathers. Oecologia 137:153–158. 12811535 10.1007/s00442-003-1307-3

[ece31910-bib-0044] Nussey, D. H. , T. Coulson , M. Festa‐Bianchet , and J. M. Gaillard . 2008 Measuring senescence in wild animal populations: towards a longitudinal approach. Funct. Ecol. 22:393–406.

[ece31910-bib-0045] Palokangas, P. , E. Korpimäki , H. Hakkarainen , E. Huhta , P. Tolonen , and R. V. Alatalo . 1994 Female kestrels gain reproductive success by choosing brightly ornamented males. Anim. Behav. 47:443–448.

[ece31910-bib-0046] Partridge, L. , and N. H. Barton . 1996 On measuring the rate of ageing. Proc. Biol. Sci. 263:1365–1371. 10.1098/rspb.1996.0113 8763795

[ece31910-bib-0047] van de Pol, M. , and S. Verhulst . 2006 Age‐dependent traits: a new statistical model to separate within‐ and between‐individual effects. Am. Nat. 167:766–773. 16671020 10.1086/503331

[ece31910-bib-0048] van de Pol, M. , and J. Wrigth . 2009 A simple method for distinguishing within‐ versus between‐subject effects using mixed models. Anim. Behav. 77:753–758.

[ece31910-bib-0049] Potti, J. , D. Canal , and D. Serrano . 2013 Lifetime fitness and age‐related female ornament signalling: evidence for survival and fecundity selection in the pied flycatcher. J. Evol. Biol. 26:1445–1457. 23638705 10.1111/jeb.12145

[ece31910-bib-0050] Potti, J. , D. Canal , and C. Camacho . 2014 Ontogenetic variation in the plumage colour of female European Pied Flycatchers *Ficedula hypoleuca* . The Ibis 156:879–884.

[ece31910-bib-0051] Rebke, M. , T. Coulson , P. H. Becker , and J. W. Vaupel . 2010 Reproductive improvement and senescence in a long‐lived bird. Proc. Natl Acad. Sci. USA 107:7841–7846. 20378836 10.1073/pnas.1002645107 PMC2867923

[ece31910-bib-0052] Rivera‐Gutierrez, H. F. , R. Pinxten , and M. Eens . 2010 Multiple signals for multiple messages: great tit, Parus major, song signals age and survival. Anim. Behav. 80:451–459.

[ece31910-bib-0053] Robinson, M. R. , G. Sander van Doorn , L. Gustafsson , and A. Qvarnström . 2012 Environment‐dependent selection on mate choice in a natural population of birds. Ecol. Lett. 15:611–618. 22487545 10.1111/j.1461-0248.2012.01780.x

[ece31910-bib-0054] Roulin, A. 1999 Nonrandom pairing by male barn owls (*Tyto alba*) with respect to a female plumage trait. Behav. Ecol. 10:688–695.

[ece31910-bib-0055] Roulin, A. 2004 Proximate basis of the covariation between a melanin‐based female ornament and offspring quality. Oecologia 140:668–675. 15248061 10.1007/s00442-004-1636-x

[ece31910-bib-0056] Roulin, A. , and C. Dijkstra . 2003 Genetic and environmental components of variation in eumelanin and phaeomelanin sex‐traits in the barn owl. Heredity 50:6. 10.1038/sj.hdy.6800260 12714980

[ece31910-bib-0057] Torres, R. , and A. Velando . 2007 Male reproductive senescence: the price of immune‐induced oxidative damage on sexual attractiveness in the blue‐footed booby. J. Anim. Ecol. 76:1161–1168. 17922712 10.1111/j.1365-2656.2007.01282.x

[ece31910-bib-0058] Vaupel, J. W. , and A. I. Yashin . 1985 Heterogeneity's ruses: some surprising effects of selection on population dynamics. Am. Stat. 39:176–185. 12267300

[ece31910-bib-0059] Velando, A. , H. Drummond , and R. Torres . 2010 Senescing sexual ornaments recover after a sabbatical. Biol. Lett. 6:194–196. 19955170 10.1098/rsbl.2009.0759 PMC2865038

[ece31910-bib-0060] Vergara, P. , and J. A. Fargallo . 2007 Delayed plumage maturation in Eurasian kestrels: female mimicry, subordination signalling or both? Anim. Behav. 74:1505–1513.

[ece31910-bib-0061] Vergara, P. , and J. Martínez‐Padilla . 2012 Social context decouples the relationship between a sexual ornament and testosterone levels in a male wild bird. Horm. Behav. 62:407–412. 22841824 10.1016/j.yhbeh.2012.07.007

[ece31910-bib-0062] Vergara, P. , J. A. Fargallo , J. Martínez‐Padilla , and J. A. Lemus . 2009 Inter‐annual variation and information content of melanin‐based coloration in female Eurasian kestrels. Biol. J. Linn. Soc. 97:781–790.

[ece31910-bib-0063] Vergara, P. , J. Martinez‐Padilla , F. Mougeot , F. Leckie , and S. M. Redpath . 2012a Environmental heterogeneity influences the reliability of secondary sexual traits as condition indicators. J. Evol. Biol. 25:20–28. 22022806 10.1111/j.1420-9101.2011.02399.x

[ece31910-bib-0064] Vergara, P. , F. Mougeot , J. Martínez‐Padilla , F. Leckie , and S. M. Redpath . 2012b The condition dependence of a secondary sexual trait is stronger under high parasite infection level. Behav. Ecol. 23:502–511.

[ece31910-bib-0065] Vergara, P. , S. M. Redpath , J. Martínez‐Padilla , and F. Mougeot . 2012c Environmental conditions influence red grouse ornamentation at a population level. Biol. J. Linn. Soc. 107:788–798.

[ece31910-bib-0066] Village, A. 1990 The kestrel. T & A. D. Poyser, London.

[ece31910-bib-0067] Williams, G. C. 1966 Natural selection, the costs of reproduction, and a refinement of Lack's principle. Am. Nat. 100:687–690.

[ece31910-bib-0068] Zahavi, A. 1975 Mate selection—A selection for a handicap. J. Theor. Biol. 53:205–214. 1195756 10.1016/0022-5193(75)90111-3

