# Corrigendum

**DOI:** 10.1002/ece3.3044

**Published:** 2017-05-16

Lopez‐Idiaquez, D., Vergara, P., Fargallo, J. A. and Martinez‐Padilla, J. (2016). Old males reduce melanin‐pigmented traits and increase reproductive outcome under worse environmental conditions in common kestrels. *Ecology and Evolution*,* 6*, 1224‐1235.

We report a typo in a *p*‐value in our results. In the first paragraph of the subsection “Environ.mental association between reproduction and ornament expression,” in the sentence that reads “We also found a marginal tendency for early life individuals (WIA_c_ < 0: −0.087 ± 0.042, *F*_1,10_ = 4.204, *p* = .067) and no significant relationship for those in age class 3 (WIA_c_ > 0: *p *= .0344).” This last *p*‐value is wrongly written, and it has to be .344 instead. Therefore, the sentence should read “We also found a marginal tendency for early life individuals (WIA_c_ < 0: −0.087 ± 0.042, *F*_1,10_ = 4.204, *p* = .067) and no significant relationship for those in age class 3 (WIA_c_ > 0: *p *= .344)”.

This is a typo and neither represents an error in our analyzes nor a wrong interpretation of our results. Thus, the discussion and the conclusions of the study remain as they are explained throughout the discussion.

