# Supplementary material for: Old males reduce melanin‐pigmented traits and increase reproductive outcome under worse environmental conditions in common kestrels
Source: Ecol Evol. 2016 Jan 27;6(4):1224–35. doi: 10.1002/ece3.1910 (PMC4761766; doi:10.1002/ece3.1910)
Supplement: Supplementary file 1 — Data S1. Inter‐relationship melanin‐based measured traits. Table S1. Results of LMM analysing the inter‐relationship of the spot number in four patches of male common kestrels (Falco tinnunculus). Table S2. Results of LMM with normal errors analysing the inter‐relationship of the spot size in four patches of male common kestrels (Falco tinnunculus). Table S3. Results of LMM analysing the inter‐relationship of the spot number in four patches of female common kestrels (Falco tinnunculus). Table S4. Results of GLMM with normal errors analysing the inter‐relationship of the spot size in four patches of female common kestrels (Falco tinnunculus). Table S5. Results of the LMM analysing the relationship between the number and size of the spots in each patch in male common kestrels (Falco tinnunculus). Table S6. Results of the LMM analysing the relationship between the number and size of the spots in each patch in female common kestrels (Falco tinnunculus). Figure S1. Common kestrel dorsum divided in four areas: (a) back, (b) auxiliary feathers (c) greater coverts and (d) median and lesser coverts. Data S2. Measured trait repeatability. Table S7. Results of the repeatability analysis done for the size of the measured traits in both males and females. Data S3. Number of observations for males and females in each age class. Table S8. Number of observations on each age class in males and females. [file ECE3-6-1224-s001.docx]

APPENDIX

Data-S1<- Inter-relationship melanin-based measured traits

Preliminarily we analysed the inter-relationship between the common kestrels plumage spots. With this aim we divided kestrels dorsum in four areas (a) back, (b) auxiliary feathers, (c) greater coverts and (d) median and lesser coverts (Fig S1). In each of them we counted the number of spots and randomly selected 5 of them to measure the size, except for the great coverts where we always measured the same spot (red circle). Then we constructed LMM, between the number of spots and the size in each area for both males and females. We included individual identity as a random effect to avoid pseudoreplication ([Hurlbert 1984](#_ENREF_1)) because we have repeated measures of the same individuals. Our results show that there is a high positive relation between the number of spots (Table S1) and the area in the four zones of males kestrel dorsum (Table S2). That is, if one male has a great number of spots in the back, it will have a lot of spots in the other patches and the same with the back spot size. In the case of the females we did not found any consistent pattern (Tables S3 and S4). We also analysed the relation between the number and size of the spots in each patch. For both males and females (Tables S5 and S6) there is not a consistent pattern in the relationship suggesting that these two variables have different influences.

|  | Number of back spots | Number of auxiliary feather spots | Number of greater covert spots | Number of median and lesser covers spots |
| --- | --- | --- | --- | --- |
| Number of back spots |  | 1.201±0.176**** | 0.658±0.234*** | 0.249±0.032**** |
| Number of auxiliary feather spots | 1.201±0.176**** |  | 0.167±0.088* | 0.087±0.012**** |
| Number of greater covert spots | 0.658±0.234*** | 0.167±0.088* |  | 0.041±0.011*** |
| Number of median and lesser covers spots | 0.249±0.032**** | 0.087±0.012**** | 0.041±0.011*** |  |

Table S1. Results of LMM analysing the inter-relationship of the spot number in four patches of male common kestrels (*Falco tinnunculus*). **P*<0.1 (Marginally significant) ***P*<0.05 ****P*<0.01*****P*<0.0001 (n=175)

|  | Back spot size | Auxiliary feather spots size | Greater covert spot size | Median and lesser covers spot size |
| --- | --- | --- | --- | --- |
| Back spot size |  | 0.254±0.038**** | 0.050±0.138*** | 0.352±0.101**** |
| Auxiliary feather spots size | 0.254±0.038**** |  | 0.106±0.024*** | 0.762±0.176*** |
| Greater covert spot size | 0.050±0.138*** | 0.106±0.024*** |  | 1.917±0.532*** |
| Median and lesser covers spot size | 0.352±0.101**** | 0.762±0.176*** | 1.917±0.532*** |  |

Table S2. Results of LMM with normal errors analysing the inter-relationship of the spot size in four patches of male common kestrels (*Falco tinnunculus*). Significant variables are in bold. **P*<0.1 (Marginally significant) ***P*<0.05 ****P*<0.01*****P*<0.0001 (n=175)

|  | Number of back spots | Number of auxiliary feather spots | Number of greater covert spots | Number of median and lesser covers spots |
| --- | --- | --- | --- | --- |
| Number of back spots |  | 0.379±0.182 | 0.002±0.341 | 0.099±0.080 |
| Number of auxiliary feather spots | 0.379±0.182 |  | 0.0533±0.202** | 0.141±0.050** |
| Number of greater covert spots | 0.002±0.341 | 0.0533±0.202** |  | 0.034±0.028 |
| Number of median and lesser covers spots | 0.099±0.080 | 0.141±0.050** | 0.034±0.028 |  |

Table S3. Results of LMM analysing the inter-relationship of the spot number in four patches of female common kestrels (*Falco tinnunculus*). **P*<0.1 (Marginally significant) ***P*<0.05 ****P*<0.01*****P*<0.0001 (n=68)

|  | Back spot size | Auxiliary feather spots size | Greater covert spot size | Median and lesser covers spot size |
| --- | --- | --- | --- | --- |
| Back spot size |  | 0.076±0.073 | 0.012±0.034 | 0.163±0.133 |
| Auxiliary feather spots size | 0.076±0.073 |  | 0.065±0.058 | 0.456±0.209** |
| Greater covert spot size | 0.012±0.034 | 0.065±0.058 |  | 1.058±0.392** |
| Median and lesser covers spot size | 0.163±0.133 | 0.456±0.209** | 1.058±0.392** |  |

Table S4. Results of GLMM with normal errors analysing the inter-relationship of the spot size in four patches of female common kestrels (*Falco tinnunculus*). **P*<0.1 (Marginally significant) ***P*<0.05 ****P*<0.01*****P*<0.0001 (n=68)

| Parameter | Estimate | SE | *F* | *P* |
| --- | --- | --- | --- | --- |
| Number of back spots (n=175) | | | | |
| Back spot size | 0.146 | 0.263 | F_1,49_=0.311 | 0.579 |
| Number of auxiliary feathers spots (n=175) | | | | |
| Auxiliary feather spot size | 0.069 | 0.055 | F_1,49_=1.566 | 0.216 |
| Number of greater coverts spots (n=175) | | | | |
| Size of greater covert spots | 0.054 | 0.015 | F_1,49_=13.258 | **0.0007** |
| Number of median and lesser coverts (n=175) | | | | |
| Size of median and lesser covert spots | 0.152 | 0.716 | F_1,49_=0.045 | 0.831 |

Table S5. Results of the LMM analysing the relationship between the number and size of the spots in each patch in male common kestrels (*Falco tinnunculus*). Significant variables are in bold.

| Parameter | Estimate | SE | *F* | *P* |
| --- | --- | --- | --- | --- |
| Number of back spots (n=68) | | | | |
| Back spot size | -0.088 | 0.337 | F_1,19_=0.068 | 0.795 |
| Number of auxiliary feather spots (n=68) | | | | |
| Auxiliary feather spot size | -0.441 | 0.122 | F_1,19=_13.055 | **0.001** |
| Number of greater coverts spots (n=68) | | | | |
| Size of greater coverts spots | 0.061 | 0.032 | F_1,19_=3.444 | 0.079 |
| Number of median and lesser coverts (n=68) | | | | |
| Size of median and lesser coverts spots | 0.676 | 0.537 | F_1,19_=1.579 | 0.224 |

Table S6. Results of the LMM analysing the relationship between the number and size of the spots in each patch in female common kestrels (*Falco tinnunculus*). Significant variables are in bold.


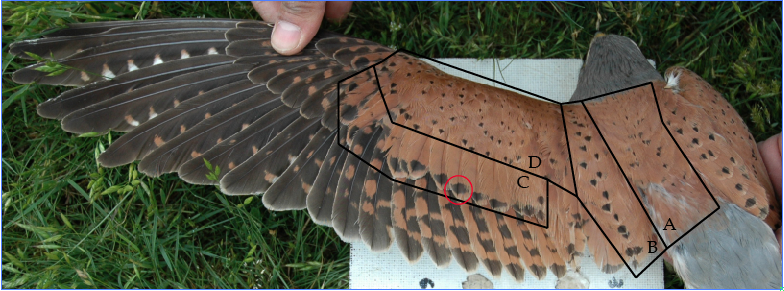


Fig. S1 Common kestrel dorsum divided in four areas: (a) back, (b) auxiliary feathers (c) greater coverts and (d) median and lesser coverts. The red circle represents the measured spot of that area.

Data-S2 <- Measured trait repeatability

|  | Males | | Females | |
| --- | --- | --- | --- | --- |
| Trait | R | P | R | P |
| Back spot size | 0.742 | **<0.0001** | 0.711 | **<0.0001** |
| Auxiliary feathers spot size | 0.606 | **<0.001** | 0.210 | 0.10 |
| Median and lesser spot size | 0.337 | **0.04** | 0.390 | **0.01** |

Table S7. Results of the repeatability analysis done for the size of the measured traits in both males and females.

We also measured the repeatability ([Lessells & Boag 1987](#_ENREF_2); [Nakagawa & Schielzeth 2010](#_ENREF_3)) of the measures that we took for both males and females. Our results indicate that only the measures of the size of back spots are repeatable enough. Considering what we stated below the number and size of both males and female common kestrel is the only melanin-based trait that we can measure efficiently. This is the main reason that we follow to use these traits in our analyses.

Data-S3<- Number of observations for males and females in each age class.

| **Age** | **2** | **3** | **4** | **5** | **6** | **7** | **8** | **9** | **Total** |
| --- | --- | --- | --- | --- | --- | --- | --- | --- | --- |
| *Males* | | | | | | | | | |
| Reproductive traits | 53 | 24 | 13 | 13 | 4 | 1 | 1 | - | 109 |
| Ornamental traits | 51 | 23 | 12 | 13 | 4 | 1 | 1 | - | 105 |
| *Females* | | | | | | | | | |
| Reproductive traits | 31 | 18 | 10 | 5 | 4 | 2 | 2 | 2 | 75 |
| Ornamental traits | 31 | 18 | 10 | 5 | 4 | 2 | 2 | 2 | 75 |

Table S8. Number of observations on each age class in males and females.

References

Hurlbert, S.H. (1984) Pseudoreplication and the Design of Ecological Field Experiments. *Ecological Monographs,* **54,** 187-211.

Lessells, C.M. & Boag, P.T. (1987) Unrepeatable repeatabilities a common mistake. *The Auk*.

Nakagawa, S. & Schielzeth, H. (2010) Repeatability for Gaussian and non-Gaussian data: a practical guide for biologists. *Biological Reviews,* **85,** 935-956.
